# Central venous catheter-related bacteremia caused by *Kocuria kristinae*: Case report and review of the literature

**DOI:** 10.1186/1476-0711-10-31

**Published:** 2011-08-24

**Authors:** Ryan Dunn, Sara Bares, Michael Z David

**Affiliations:** 1 Department of Medicine, University of Chicago, Chicago, Illinois, USA; 2 Section of Infectious Diseases and Global Health, Department of Medicine, University of Chicago, Chicago, Illinois, USA; 3 Section of Infectious Diseases and Global Health, Department of Medicine, University of Chicago, Chicago, Illinois, USA

## Abstract

*Kocuria *species are unusual human pathogens isolated most commonly from immunocompromised hosts, such as transplant recipients and cancer patients undergoing chemotherapy, or from patients with chronic medical conditions. A case of catheter-related bacteremia with pulmonary septic emboli in a pregnant adult female without chronic medical conditions is described. A review of other reported *Kocuria *infections is provided.

## Introduction

*Kocuria *species are ubiquitous in the environment and part of normal skin and oral flora of humans and other mammals [1]. They are, however, uncommon human pathogens with only a limited number of cases reported in the literature. *K. kristinae *is a facultative anaerobic bacterium that is a non-motile, catalase-positive, coagulase-negative, gram- positive coccus that occurs in tetrads. It has been previously reported to cause bacteremia in chronically ill patients with malignancies or other immunosuppressed states [2-4]. *K. kristinae *has been associated with one case of cholecystitis [5]. Recently, it has been associated with two cases of peritonitis related to peritoneal dialysis [6,7]. To the best of our knowledge we present here the first reported case of *K. kristinae *bacteremia in a pregnant, but otherwise healthy adult female.

## Case Presentation

In August 2010, a 29-year-old African American female who was 16 weeks pregnant presented to her primary care physician complaining of a fever of 103 degrees Fahrenheit, chills, pleuritic chest pain, shortness of breath and a non-productive cough for 2 days. Her pregnancy had been complicated by thyrotoxicosis and hyperemesis gravidarum requiring the placement of a peripherally inserted central venous catheter for total parenteral nutrition (TPN). The catheter had been placed approximately 11 days prior to the onset of her symptoms and she had received TPN since that time. The patient had no other significant medical history and was born in the United States with no recent travel or unusual food exposures. She did not smoke tobacco, drink alcohol or use illicit drugs. She was sexually active with a single male partner and had no history of sexually transmitted diseases. Her only sick contact was her father who had recently been treated for cellulitis. Her immunization history was unknown.

The patient was initially given a 5-day course of azithromycin. No laboratory studies or cultures were performed at the time. Three days later she returned to the emergency department of a community hospital complaining of persistent symptoms and mild vaginal spotting. She continued to have fevers, chills, pleuritic chest pain and dyspnea that had improved minimally with azithromycin.

A chest x-ray revealed diffuse, peripherally located reticulonodular infiltrates in both lung fields. Laboratory testing demonstrated an elevated white blood cell count (15,800 cells per cubic millimeter). The automated differential showed 90% neutrophils and 6% bands. A complete metabolic panel was within normal limits. Her albumin, a general marker of nutritional status, was within normal range. The patient was admitted to the hospital. Two sets of blood cultures, both peripheral and from her central venous catheter, were drawn at the time of admission. Both sets had bacterial growth (at 44 and 30 hours of incubation, respectively) with gram-positive cocci in clusters reported on gram stain. A rapid influenza antigen test and HIV ELISA were negative.

Ceftriaxone and azithromycin were administered for community-acquired pneumonia. Vancomycin and oseltamivir were added on day 1 of hospitalization because there was concern for both methicillin-resistant *Staphylococcus aureus *pneumonia and a preceding influenza-like illness. Surveillance cultures of the blood were drawn daily. These remained negative after antibiotic therapy was initiated.

**Figure 1:**
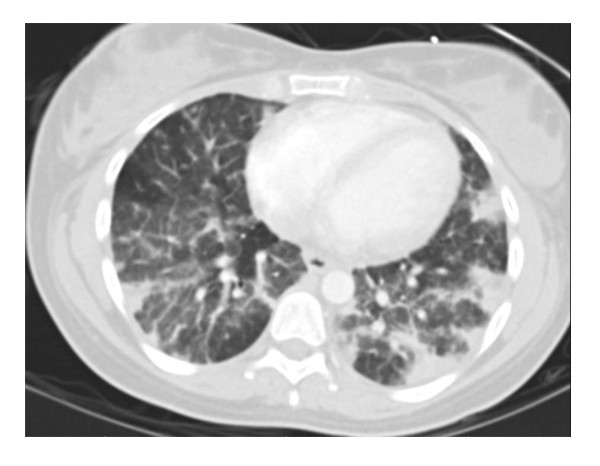
**Computed tomography scan of the chest performed on hospital day three revealing bilateral, peripheral reticulonodular opacities consistent with septic pulmonary emboli**.

The patient continued to have persistent symptoms after the initiation of antibiotic therapy. She required no supplemental oxygen and had no hemodynamic compromise, but she remained intermittently febrile and dyspneic. Her vaginal spotting resolved. On day 2 of hospitalization the microbiology laboratory identified the organism from the blood as presumed *Staphylococcus *species based on gram stain. The central venous catheter was removed on hospital day 3 and the catheter tip was cultured but had no bacterial growth. Computed tomography (CT) of the chest revealed bilateral peripheral, reticular nodular densities consistent with septic emboli (see Figure 1.). An upper extremity ultrasound revealed a thrombosis in the right brachial vein. Heparin was initiated for anticoagulation and intravenous clindamycin was added to broaden the antibiotic regimen. The patient was transferred to the University of Chicago Medical Center (UCMC) for further care on hospital day 4. A transesophageal echocardiogram was obtained upon transfer and revealed no valvular vegetations. Azithromycin, oseltamivir and ceftriaxone were discontinued, and vancomycin and clindamycin were continued.

Four days after cultures were drawn, the organism isolated, initially presumed to be *Staphylococcus *species, was speciated as *K. kristinae *by an automated system (Vitek-2, La Balme les Grottes, France). The peripheral cultures grew the bacterium in both the aerobic and anaerobic bottles while the central venous catheter cultures grew in the aerobic bottle only. The presence of multiple positive cultures from different sites suggested that contamination was not likely. Because this organism was unusual, the isolates were sent to the Mayo Medical Laboratories, and there 16s RNA gene sequencing confirmed the isolate as *K. kristinae*. The organism was catalase positive, coagulase negative and PYR positive. Interestingly, the Staphaurex latex bead agglutination test (Remel, Lenexa, Kansas) was also positive. This test identifies the presence of clumping factor and protein A produced by most strains of *S. aureus*.

Antimicrobial susceptibilities were performed on the Vitek-2 automated system at both institutions and the *K. kristinae *isolate was found to be susceptible to oxacillin, vancomycin, cefazolin, clindamycin, rifampin and trimethoprim/sulfamethoxazole.

Septic emboli seen on CT were felt to be secondary to septic thrombosis complicating a catheter-related bloodstream infection. The patient's pleuritic chest pain and dyspnea improved gradually although she remained intermittently febrile, with a temperature of 100.6 on hospital days 8 and 9 (days 4 and 5 at UCMC). The patient's leukocytosis resolved after hospital day 14. When the final susceptibility testing returned on hospital day 12 (Day 8 at UCMC), oxacillin was started and vancomycin and clindamycin were discontinued. The patient continued to improve and was discharged home on hospital day 16 to complete a 4-week course of intravenous oxacillin for complicated bacteremia with enoxaparin for anticoagulation for a duration of 6 months. Upon follow up in the infectious disease clinic approximately 6 weeks after discharge the patient was doing well clinically. She had no fever, cough, shortness of breath or chest pain.

## Discussion

*K. kristinae *is a facultative anaerobic bacterium that is catalase-positive, coagulase-negative, non-motile, gram-positive, nitrite reduction negative, and esculin hydrolysis positive that occurs in tetrads and produces pale cream non-hemolytic colonies on blood agar. *Kocuria *species are members of the Micrococcus family and include *K. kristinae, K. rosea, K. varians, K. palustris, K. marina, K. aegyptia*, and *K. rhizophila *[8]. Most *Kocuria *species, with the exception of *K. kristinae*, are strict aerobes [8]. They have been identified as common skin and oral flora and have also been found in salted meats [9]. *K. kristinae *is a rare cause of human disease. We reviewed all the English and French language literature on human *Kocuria *infections reported in Medline from the time of its description as a genus in 1995 to 2010 and identified 15 cases (Table 1). In the limited number of cases reported in the literature there is no clear gender predominance and the mean age of the patients was 54 years (range 2-89). *Kocuria *species have been responsible for infections most commonly in immunocompromised hosts. In one case series, *Kocuria *species, including *K. kristinae*, were responsible for endocarditis and central venous catheter-related bacteremia exclusively among immunocompromised hosts receiving TPN [2]. All of these patients had underlying solid tumor malignancy or short gut syndrome. *K. kristinae *central venous catheter-related bacteremia has been reported in a patient with ovarian cancer undergoing chemotherapy with febrile neutropenia and in a patient with acute leukemia [3,4]*K. kristinae *was also reported to be responsible for a case of cholecystitis in an immunocompetent host as well as 2 cases of peritoneal dialysis-related peritonitis [5-7]. Other *Kocuria *species including *K. rhizophila, K. rosea, K. varians *and *K. marina *have been reported as etiologic agents in various infections including a brain abscess in a diabetic patient, central venous catheter-related bacteremia in a pediatric patient with methylmalonic aciduria, central venous catheter-related bacteremia in patient undergoing stem cell transplantation and 3 cases of peritonitis associated with peritoneal dialysis [10-14].

**Table 1:** *Kocuria *Cases Reported in the Literature

*Kocuria spp*.	Age (years)	Gender	Site of Isolation	Country	Medical condition or underlying disease	Reference
*K. kristinae*	2	male	blood	China	Congenital short bowel, hypogammaglobulinemia, central venous catheter for TPN	[2]

*K. kristinae*	29	female	blood	United States	Pregnancy, hyperemesis gravidarum, central venous catheter for TPN	Current report

*K. kristinae*	89	female	blood, endocarditis	China	Ischemic bowel status post resection, short bowel syndrome, central venous catheter for TPN	[2]

*K. kristinae*	37	female	blood	China	Gastric cancer, central venous catheter for TPN	[2]

*K. kristinae*	68	female	blood	China	Gastric cancer, central venous catheter for TPN	[2]

*K. kristinae*	51	female	blood	Italy	Ovarian cancer undergoing chemotherapy, permanent central venous catheter	[3]

*K. kristinae*	56	male	biliary fluid	Hong Kong	Gallstones	[5]

*K. kristinae*	68	male	blood	France	MDS, acute myelogenous leukemia, tuberculosis, central venous catheter for chemotherapy	[4]

*K. kristinae*	78	male	peritoneal fluid	Italy	End-stage renal disease on chronic ambulatory peritoneal dialysis	[7]

*K. kristinae*	69	male	peritoneal fluid	China	End-stage renal disease on chronic ambulatory peritoneal dialysis	[6]

*K. marina*	57	male	peritoneal fluid	South Korea	End-stage renal disease on chronic ambulatory peritoneal dialysis	[11]

*K. marina*	73	male	peritoneal fluid	South Korea	End-stage renal disease on chronic ambulatory peritoneal dialysis	[11]

*K. rhizophila*	8	male	blood	Germany	Methylmalonic aciduria, pancreatic pseudocyst, central venous catheter for TPN	[12]

*K. rosea*	39	male	blood, catheter tip	Turkey	Central venous catheter-related infection, Hodgkin's disease, undergoing peripheral blood stem cell transplantation	[13]

*K. rosea*	56	female	peritoneal fluid	Turkey	End-stage renal disease on chronic ambulatory peritoneal dialysis	[14]

*K. varians*	52	male	central nervous system	China	Diabetes mellitus	[10]

TPN, total parenteral nutrition.

MDS, myelodysplastic syndrome

Bacteremia caused by *K. kristinae *in an immunocompetent host such as the pregnant but otherwise healthy 29 year-old female in our case has not been reported to the best of our knowledge. While pregnancy does predispose to other types of severe infections and thrombus formation, it is unknown if it was a predisposing factor in this case. The complicated bacteremia resulting in septic emboli to the lungs was striking.

As in our patient, TPN administration was frequently observed in the cases of *K. kristina*e central venous catheter-related bacteremia [2]. This suggests that administration of TPN, in addition to central venous catheter placement, may be a possible risk factor for an infection with this organism.

Interestingly the organism in the present case was initially presumed to be a *Staphylococcus *species at the community hospital but was later confirmed to be *K. kristinae *by both the automated identification system at the original hospital and the reference laboratory where sequencing was performed. While there is a possibility of misidentification of these organisms using automated systems such as the Vitek-2 this may be less likely with recent versions of this system [15,16]. Furthermore, confirmatory 16s rRNA sequencing performed at the reference laboratory makes misidentification in this instance highly unlikely. The positive Staphaurex test was curious as this is a generally a specific test for *S. aureus*. The significance of this is unknown and was not described in any other reported cases.

Final identification as *K. kristinae *was performed at the Mayo Medical Laboratories with 16s RNA sequencing. The MIC values for the isolate were as follows: cefazolin = 2, rifampin < 0.5, clindamycin < 0.5, vancomycin = 2, trimethoprim/sulfamethoxazole < 0.5/9.5, oxacillin < 0.25. The breakpoints that were used were those used with *Staphylococcus *species and the organism was considered susceptible to all antibiotics tested. In contrast to our findings, Lai et al. reported *K. kristinae *isolates with an MIC to oxacillin as high as 4, which would be considered resistant for *S. aureus/S. lugdunensis *and coagulase-negative *Staphylococcus *species [2]. In the other reported cases of *K. kristinae *infections, the organisms were reported to be susceptible to many commonly used antibiotics including penicillins, macrolides, clindamycin, trimethoprim/sulfamethoxazole, vancomycin and fluoroquinolones [2,3,5].

While there are no evidence-based guidelines for the management of this uncommon infection, previous cases have been managed successfully with a number of different antimicrobial drug therapies. Monotherapy with oxacillin, vancomycin, piperacillin/tazobactam and ciprofloxacin and combination therapy with teicoplanin and vancomycin, ciprofloxacin and clindamycin as well as ceftriaxone and ofloxacin have been used successfully in case reports [2-4]. Previous reports have suggested that removal of the intravascular catheter should be considered and may be necessary for cure in cases of central venous catheter-associated bacteremia [2,3].

In conclusion, this is the first reported case of *K. kristinae *bacteremia occurring in an immunocompetent host causing a severe intravascular infection complicated by suppurative thrombosis and septic pulmonary emboli. This case expands the clinical spectrum of disease caused by these unusual pathogens and adds to the growing body of literature documenting the pathogenicity of these organisms in humans.

## Consent

Written informed consent was obtained from the patient for publication of this case report and any accompanying images. A copy of the written consent is available for review by the Editor-in-Chief of this journal.

## Competing interests

The authors declare that they have no competing interests.

## Authors' contributions

RD was the primary author for the manuscript. MD assisted in editing the manuscript. SB assisted in data gathering and editing of the manuscript. All authors made substantial contributions to the acquisition of data. All authors read and approved the final manuscript prior to publication.

## References

[B1] Szczerba I Occurrence and number of bacteria from the *Micrococcus*, *Kocuria*, *Nesterenkonia*, *Kytococcus *and *Dermacoccus *genera on skin and mucous membranes in humans Med Dosw Mikrobiol 2003 55 67 74 Polish 12908417

[B2] Lai CC Wang JY Lin SH Tan CK Wang CY Liao CH Chou CH Huang YT Lin HI Hsueh PR Catheter-related bacteraemia and infective endocarditis caused by *Kocuria *species Clin Microbiol Infect 2011 17 190 2 10.1111/j.1469-0691.2010.03211.x 20218989

[B3] Basaglia G Carretto E Barbarini D Moras L Scalone S Marone P De Paoli P Catheter-related bacteremia due to *Kocuria kristinae *in a patient with ovarian cancer J Clin Microbiol 2002 40 311 3 10.1128/JCM.40.1.311-313.2002 11773142 PMC120093

[B4] Martinaud C Gaillard T Brisou P Gisserot O de Jaureguiberry JP Bacteremia caused by *Kocuria kristinae *in a patient with acute leukemia Med Mal Infect 2008 38 165 6 French 10.1016/j.medmal.2007.11.006 18395377

[B5] Ma ES Wong CL Lai KT Chan EC Yam WC Chan AC *Kocuria kristinae *infection associated with acute cholecystitis BMC Infec Dis 2005 5 60 10.1186/1471-2334-5-60 PMC1181815 16029488

[B6] Cheung C Cheng N Chau C Li CS An unusual organism for CAPD-related peritonitis: Kocuria kristinae Perit Dial Int 2011 31 107 8 10.3747/pdi.2010.00125 21282396

[B7] Calini A Mattei R Lucarotti I Bartelloni A Rosati A *Kocuria kristinae*: an unusual cause of acute peritoneal dialysis-related infection Perit Dial Int 2011 31 105 7 21282394 10.3747/pdi.2010.00132

[B8] Stackebrandt E Kock C Gvozdiak O Schumann P Taxonomic dissection of the genus *Micrococcus*: Kocuria gen. nov., *Nesterenkonia *gen. nov., *Kytococcus *gen. nov., *Dermacoccus *gen. nov., and *Micrococcus *Cohn 1872 gen. emend Int J Syst Bacteriol 1995 45 682 92 10.1099/00207713-45-4-682 7547287

[B9] Cordero MR Zumalacárregui JM Characterization of *micrococcaceae *isolated from salt used for Spanish dry-cured ham Lett Appl Microbiol 2000 31 303 6 10.1046/j.1472-765x.2000.00818.x 11068912

[B10] Tsai CY Su SH Cheng YH Chou YL Tsai TH Lieu AS *Kocuria varians *infection associated with brain abcess: A case report BMC Infect Dis 2010 10 102 10.1186/1471-2334-10-102 20423506 PMC2875226

[B11] Lee JY Kim SH Jeong HS Oh SH Kim HR Kim YH Lee JN Kook JK Kho WG Bae IK Shin JH Two cases of peritonitis caused by *Kocuria marina *in patients undergoing continuous ambulatory peritoneal dialysis J Clin Microbiol 2009 47 2276 8 10.1128/JCM.00847-09 PMC2756933 19692561

[B12] Becker K Rutsch F Uekötter A Kipp F König J Marquardt T Peters G von Eiff C *Kocuria rhizophila *adds to the emerging spectrum of *micrococcal *species involved in human infections J Clin Microbiol 2008 46 3537 9 10.1128/JCM.00823-08 18614658 PMC2566074

[B13] Altuntas F Yildiz O Eser B Gundogan K Sumerkan B Cetin M Catheter-related bacteremia due to *Kocuria rosea *in a patient undergoing peripheral blood stem cell transplantation BMC Infect Dis 2004 4 62 10.1186/1471-2334-4-62 15615593 PMC545057

[B14] Kaya KE Kurtoglu Y Cesur S Bulut C Kinikli S Irmak H Demiröz AP Karakoç E Peritonitis due to *Kocuria rosea *in a continuous ambulatory peritoneal dialysis case Mikrobiyol Bul 2009 43 335 7 19621623

[B15] Ben-Ami R Navon-Venezia S Schwartz D Schlezinger Y Mekuzas Y Carmeli Y Erroneous reporting of coagulase-negative *Staphylococci *as *Kocuria spp*by the Vitek 2 system. J Clin Microbiol 2005 43 1448 50 10.1128/JCM.43.3.1448-1450.2005 PMC1081215 15750130

[B16] Boudewijns M Vandeven J Verhaegen J Ben-Ami R Carmeli Y Vitek 2 automated identification system and *Kocuria kristinae* J Clin Microbiol 2005 43 5832 10.1128/JCM.43.11.5832.2005 16272536 PMC1287845

